# 77 How do we include low SES girls in sport? A qualitative study on girls’ perceived barriers to sports participation

**DOI:** 10.1093/eurpub/ckae114.076

**Published:** 2024-09-26

**Authors:** Cecilie Karen Ljungmann, Julie Hellesøe Christensen, Helene Rald Johnsen, Charlotte Klinker, Charlotte Pawlowski

**Affiliations:** University Of Southern Denmark, Denmark

## Abstract

**Purpose:**

Girls are more likely to drop out of sport during adolescence than boys, in particular girls from low SES neighbourhoods. It is important to listening to these girls to get knowledge to do inclusive actions to increase low SES girls in sport. However, no studies have investigated barriers to sports participation among girls from low SES neighbourhoods. The aim of the study was to investigate perceived barriers to sports participation among adolescent girls from low SES neighbourhoods.

**Methods:**

Eleven focus groups were conducted in five low SES neighbourhoods in Denmark from July-October 2021, involving in total 44 adolescent girls from Grade 6-9 (aged 10-16 years) who were not engaged in organised sports. Ten girls had Danish ethnic origin. Thirty-four girls had other ethnic minority background (i.e., from Afghanistan, Iraq, Lebanon, Morocco, Palestine, Somalia, Syria, or Turkey). A thematic analysis was used to analyse the girls’ perceptions managed in NVivo 12.

**Results:**

The girls prioritised homework, household duties, and socialising with friends over sports. Also, the girls refrained from participating in sport due to fear of not fitting in because of ethnic background and reluctance to participate alone. Moreover, feelings of inadequacy in sporting abilities, bodily discomfort, and negative body image were perceived as barriers. Finally, gender stereotypes, depicting sports as unsuitable for girls, along with negative attitudes from boys, further discouraged the girls’ engagement.

**Conclusions:**

The findings illustrate that barriers to sports participation among this group of girls are complex and call for multifaceted actions to address the disparities such as offering programs that are more flexible may be fruitful to address these girls’ concerns about educational commitments. A less competitive environment with more emphasis on fun, enjoyment, and inclusion, may address the feeling of lacking sporting abilities. Also, peer-leaders or a buddy system may increase a sense of belonging. These findings will be useful in order to shed light on how interventions could be designed and implemented to include low SES girls in sports communities.

**Support/Funding Source:**

The study was supported by the Novo Nordisk Foundation [grant number NNF20SH00].

